# Between-Country Inequalities in the Neglected Tropical Disease Burden in 1990 and 2010, with Projections for 2020

**DOI:** 10.1371/journal.pntd.0004560

**Published:** 2016-05-12

**Authors:** Wilma A. Stolk, Margarete C. Kulik, Epke A. le Rutte, Julie Jacobson, Jan Hendrik Richardus, Sake J. de Vlas, Tanja A. J. Houweling

**Affiliations:** 1 Department of Public Health, Erasmus MC, University Medical Center, Rotterdam, the Netherlands; 2 Center for Tobacco Control Research and Education, University of California, San Francisco, San Francisco, California, United States of America; 3 Bill & Melinda Gates Foundation, Seattle, Washington, United States of America; Centers for Disease Control and Prevention, UNITED STATES

## Abstract

**Background:**

The World Health Organization (WHO) has set ambitious time-bound targets for the control and elimination of neglected tropical diseases (NTDs). Investing in NTDs is not only seen as good value for money, but is also advocated as a pro-poor policy since it would improve population health in the poorest populations. We studied the extent to which the disease burden from nine NTDs (lymphatic filariasis, onchocerciasis, schistosomiasis, soil-transmitted helminths, trachoma, Chagas disease, human African trypanosomiasis, leprosy, visceral leishmaniasis) was concentrated in the poorest countries in 1990 and 2010, and how this would change by 2020 in case the WHO targets are met.

**Principal Findings:**

Our analysis was based on 1990 and 2010 data from the Global Burden of Disease (GBD) 2010 study and on projections of the 2020 burden. Low and lower-middle income countries together accounted for 69% and 81% of the global burden in 1990 and 2010 respectively. Only the soil-transmitted helminths and Chagas disease caused a considerable burden in upper-middle income countries. The global burden from these NTDs declined by 27% between 1990 and 2010, but reduction largely came to the benefit of upper-middle income countries. Achieving the WHO targets would lead to a further 55% reduction in the global burden between 2010 and 2020 in each country income group, and 81% of the global reduction would occur in low and lower-middle income countries.

**Conclusions:**

The GBD 2010 data show the burden of the nine selected NTDs in DALYs is strongly concentrated in low and lower-middle income countries, which implies that the beneficial impact of NTD control eventually also largely comes to the benefit of these same countries. While the nine NTDs became increasingly concentrated in developing countries in the 1990–2010 period, this trend would be rectified if the WHO targets were met, supporting the pro-poor designation.

## Author Summary

The World Health Organization (WHO) has set ambitious time-bound targets for the control and elimination of neglected tropical diseases (NTDs). Investing in NTDs is seen as good value for money and as a pro-poor policy. We analyzed 1990 and 2010 burden estimates from the Global Burden of Disease Study 2010 for nine selected NTDs. These data show that the NTD disease burden is strongly concentrated in low and lower-middle income countries in 1990 and 2010. The global burden from these nine NTDs declined by 27% between 1990 and 2010, but the reduction was only 6% in the low income countries compared to 56% in upper-middle income countries, explaining the trend of increasing concentration of the burden in the poorest countries. This trend would be rectified if the WHO targets were met, supporting the pro-poor designation of public policies against NTDs.

## Introduction

The term neglected tropical diseases (NTDs) is used to denote a diverse group of infectious diseases, which are mostly confined to (sub)tropical resource-constrained regions. Resources allocated towards their treatment, control and elimination have been inadequate. In spite of major advances in science, technology, and medicine, these diseases are still causing a high disease burden [1, 2]. The concentration of NTDs in (sub-)tropical resource-constrained regions is caused by climatic factors in combination with poverty-associated factors that favor the spread of the diseases and prevent adequate access to prevention and care. This explains why NTDs are also viewed as diseases of poverty [3].

NTDs have become less neglected in the past decade, following increased recognition of their high disease burden and poverty-perpetuating impact, and awareness that medicines and other interventions to fight these diseases are available but largely inaccessible to populations in need. Thanks to donations of medicines from the pharmaceutical industry, together with investments from several other organizations, NTD control has entered a new phase [4, 5]. The World Health Organization (WHO) has set ambitious targets for the control and elimination of NTDs by 2020 [6]. Broad international commitment to do what is needed to achieve these goals was expressed through the adoption of World Health Assembly Resolution on Neglected Tropical Diseases (WHA66.12 [7]), and the 2014 Addis Ababa NTD Commitment by African endemic countries [8]. By endorsing the London Declaration on Neglected Tropical Diseases 2012, pharmaceutical companies, donors, endemic country governments and non-governmental organizations involved in NTD control created a public-private partnership to ensure the necessary supplies of medicines and other interventions for the 10 diseases benefitting most from immediate targeted action [9]. For five London Declaration NTDs (lymphatic filariasis, onchocerciasis, soil-transmitted helminths, schistosomiasis and trachoma) rapid impact could be achieved by the expansion of preventive chemotherapy programmes, while for four other diseases (leprosy, human African trypanosomiasis, Chagas disease, and visceral leishmaniasis) the main impact should come from improved accessibility and individual case management, often in combination with other interventions. For Guinea worm (dracunculiasis), the 10^th^ disease, it is critical that case detection and containment is sustained to push its eradication. All this helped endemic countries to accelerate their efforts in the control and elimination of NTDs, moving towards universal access to preventive interventions and essential health services.

According to the GBD 2010 study, the 10 diseases covered by the London Declaration together accounted for about 16 million DALYs and for about 63% of the overall NTD disease burden [2]. Meeting the 2020 targets for the London Declaration NTDs will have a major health impact. We estimated that this would avert about 230 million DALYs from 2011–2020 and another 363 million DALYs between 2021–2030, through the prevention of disability and premature death [10]. Guinea worm was ignored in these calculations, since it is already close to eradication and was not covered by the GBD 2010 study. The associated economic impact of this is also high [11, 12]. Investing in NTDs is good value for money [13, 14].

NTD control is also advocated as a pro-poor policy, because it improves the health of poorest countries and the poorest groups within countries. Gaps in health between poorer and richer countries and between the poorest and richest groups within countries have been documented extensively [15]. NTDs are exemplary for the unequal distribution of health. WHO defines health equity as the absence of avoidable or remediable differences among groups of people, whether those groups are defined socially, economically, demographically or geographically. Inequities exist both within and across nations. Health equity is an explicit concern of public health policy, with the Commission of Social Determinants of health stating: “Where systematic differences in health are judged to be avoidable by reasonable action they are, quite simply, unfair. It is this that we label health inequity. Putting right these inequities–the huge and remediable differences in health between and within countries–is a matter of social justice” [15]. NTD control alone cannot solve these inequities, which are largely driven by social, economic and political environments, but it presents a step in the right direction.

As a first step to assessing the impact of current NTD control initiatives on health equity, we study how the burden of disease from the London Declaration NTDs (excluding Guinea worm) varies between countries grouped by income level and how the burden and distribution over income groups have changed between 1990 and 2010. We also look at expectations for the year 2020. This analysis is based on Global Burden of Disease (GBD) data for 1990 and 2010, and extrapolations/estimations up to 2020.

## Methods

### Burden of disease data

We expressed the burden of disease in terms of disability-adjusted life years (DALYs). The DALY is a measure of overall disease burden, which sums the number of years of life lost (YLL) due to premature mortality and the years lived with disability (YLD) due to clinical manifestations of infection (sequelae) weighted for severity [16].

Country- and disease-specific DALY estimates for the years 1990 and 2010 were directly obtained from the Global Burden of Disease 2010 (GBD 2010) study, for 183 countries [1]. Five NTDs are associated with premature mortality, i.e. human African trypanosomiasis, visceral leishmaniasis, Chagas disease, ascariasis (as one of the three soil-transmitted helminthiases) and schistosomiasis. The other diseases (lymphatic filariasis, onchocerciasis, hookworm and trichuriasis (the two other soil-transmitted helminthiases considered), trachoma, and leprosy) are associated with YLD only. Data sources, methods and assumptions used to estimate trends in the burden of disease towards 2020 and beyond are described in detail by De Vlas et al. [10]. Here, we briefly summarize the general approach and main assumptions.

The trend in expected burden after 2010 was estimated under the assumption that the WHO targets for control and elimination of these NTDs would be met. An overview of the WHO targets and our interpretation in terms of country-specific achievements is provided by in supplementary file, S1 Table. First, we studied the time-bound WHO roadmap targets to determine what the endpoint exactly entails in terms of incidence and prevalence of NTD-caused clinical manifestations (sequelae, in GBD terminology) at specific time points. In doing so, we were advised by experts from the WHO and other disease experts. For some NTDs, the target entails a reduction to zero of the incidence of infection and/or associated sequelae at a future point in time (trachoma, lymphatic filariasis, onchocerciasis, schistosomiasis). For other NTDs, it implies a reduction in the number of cases to low levels but not elimination, so that some cases of infection and associated reversible and irreversible sequelae would still occur after the targets are met (e.g. visceral leishmaniasis, Chagas disease, leprosy, soil-transmitted helminthiases, human African trypanosomiasis). The targets can be expressed in terms of incidence of clinical manifestations, but burden estimates are based on prevalence estimates. For reversible sequelae, i.e. if patients recover due to treatment or intervention within a relatively short period of time (within a couple of years at most), trends in incidence and prevalence are expected to be largely similar. However, for irreversible diseases (like blindness through onchocerciasis or trachoma) a decline in incidence is not immediately reflected in prevalence, since the latter measure will include people who acquired the clinical manifestations at some point in the past and cannot be cured. For irreversible diseases, the target was defined in terms of incidence density rates and prevalence was estimated in a second step. The year in which the targets are expected to be achieved can vary between countries. We used existing estimates from WHO and disease control programmes if available (e.g. from the WHO roadmap report and disease control programme estimates [6]) and otherwise determined the target year in discussions with experts.

We assumed that the prevalence (for reversible sequelae) and incidence (for irreversible sequelae) follow a linear decline from their levels in 2010 to their time-bound endpoint. From this, we calculated the number of prevalent cases, YLD, and YLL. DALY estimates by NTD, country, and calendar year were obtained by summing YLL and YLD estimates over sequelae and age-sex groups. See De Vlas et al [10] for a more detailed description of the methods used to estimate the number of prevalent cases at each point in time for reversible diseases, and the calculation of DALYs.

### Classification of countries according to region and income per capita

**Fig 1:**
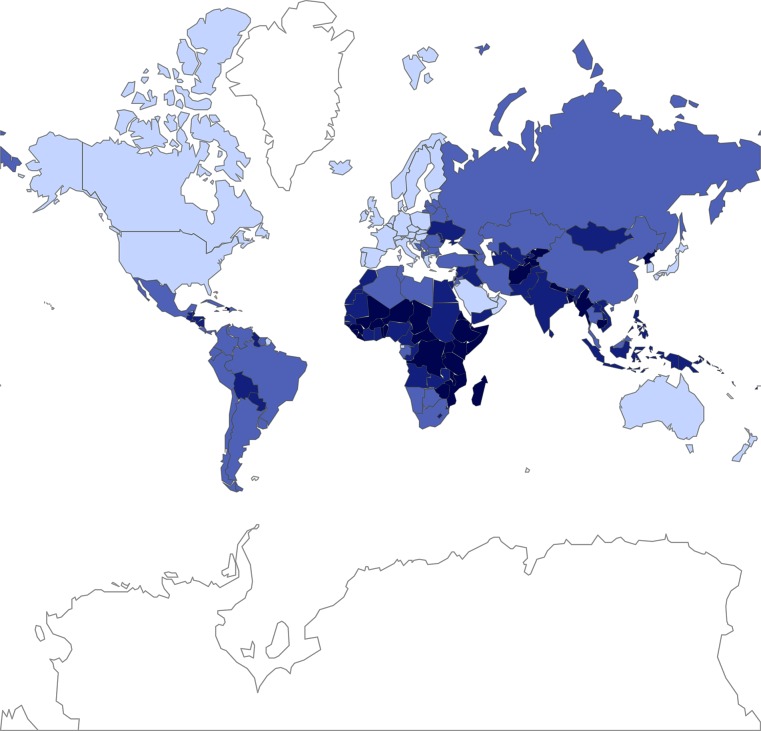
World map showing the GNI classification of countries in 2010.

The countries considered are grouped into 6 regions, as identified by WHO: Region of the Americas, South-East Asia Region, European Region, Eastern Mediterranean Region, and Western Pacific Region. See the WHO website for further information [17]. We further classified countries into four income groups, based on their Gross National Income (GNI) per capita (Atlas Method) in 2010, using the 2010 World Bank classification (Fig 1) [18]. Low income countries are those with a GNI of 1,005 US$ or less. Lower-middle and upper-middle income countries had a GNI of 1,006–3,975 US$ and 3,976–12,275 US$, respectively. Countries with a GNI of 12,276 US$ or more are classified as high-income countries. Low and lower-middle income countries are considered to be developing economies. Table 1 gives the total population (summed over countries) by income group and WHO region; in 2010, 12% of the global population lived in low income countries, 36% in lower-middle countries, and another 36% in upper-middle income countries. In Africa and South-East Asia, respectively 89% and 96% of the population lived in low or lower-middle income countries.

**Table 1:** Global population in millions in 1990, 2010 and 2020 by WHO region and 2010-GNI classification of countries.

		GNI classification 2010				
Year	WHO region	Low income	Lower-middle income	Upper-middle income	High income	Grand Total
**1990**	Africa	267.2 (53.1%)	167.5 (33.3%)	67.9 (13.5%)	0.4 (0.1%)	503.0 (100%)
	Americas	7.1 (1.0%)	35.2 (4.9%)	396.2 (54.8%)	283.9 (39.3%)	722.4 (100%)
	Eastern Mediterranean	18.1 (4.9%)	254.5 (68.7%)	74.8 (20.2%)	22.9 (6.2%)	370.2 (100%)
	Europe	9.7 (1.1%)	91.3 (10.7%)	294.7 (34.6%)	454.8 (53.5%)	850.5 (100%)
	South-East Asia	187.8 (14.3%)	1066.1 (81.3%)	56.8 (4.3%)	0 (0%)	1310.7 (100%)
	Western Pacific	9.1 (0.6%)	143.1 (9.4%)	1183.6 (77.6%)	189 (12.4%)	1524.7 (100%)
	**Total**	**499.0 (9.4%)**	**1757.7 (33.3%)**	**2074.0 (39.3%)**	**950.9 (18.0%)**	**5281.6 (100%)**
**2010**	Africa	469.9 (55.5%)	280.9 (33.2%)	95.5 (11.3%)	0.7 (0.1%)	847.1 (100%)
	Americas	9.9 (1.1%)	51.7 (5.5%)	527.1 (56.3%)	348.3 (37.2%)	937.1 (100%)
	Eastern Mediterranean	38.0 (6.6%)	394.6 (68.1%)	101.9 (17.6%)	44.5 (7.7%)	579.0 (100%)
	Europe	13.0 (1.4%)	93.8 (10.4%)	304.0 (33.7%)	491.8 (54.5%)	902.6 (100%)
	South-East Asia	254.4 (14.2%)	1468.9 (82.1%)	66.7 (3.7%)	0 (0%)	1790.0 (100%)
	Western Pacific	14.4 (0.8%)	200.6 (11.1%)	1388.1 (76.6%)	208.1 (11.5%)	1811.1 (100%)
	**Total**	**799.6 (11.6%)**	**2490.4 (36.3%)**	**2483.5 (36.2%)**	**1093.4 (15.9%)**	**6876.9 (100%)**
**2020**	Africa	618.5 (56.6%)	366.1 (33.5%)	107.0 (9.8%)	0.9 (0.1%)	1092.6 (100.0%)
	Americas	11.3 (1.1%)	61.5 (6.0%)	581.2 (56.3%)	377.6 (36.6%)	1031.7 (100.0%)
	Eastern Mediterranean	48.5 (7.0%)	470.7 (68.2%)	115.7 (16.8%)	55.5 (8.0%)	690.3 (100.0%)
	Europe	15.8 (1.7%)	96.0 (10.4%)	308.8 (33.4%)	505.3 (54.6%)	925.9 (100.0%)
	South-East Asia	281.5 (14.1%)	1647.2 (82.5%)	68.2 (3.4%)	0.0 (0.0%)	1996.9 (100.0%)
	Western Pacific	16.9 (0.9%)	229.0 (11.9%)	1465.7 (76.2%)	212.9 (11.1%)	1924.6 (100.0%)
	**Total**	992.4 (13.0%)	2870.5 (37.5%)	2646.7 (34.5%)	1152.3 (15.0%)	7662.0 (100.0%)

### Availability of data and materials

All data underlying the analysis are provided in S1 Datafile. The resulting health impact and the NTD-specific assumptions are also available as an open-access web-based dissemination tool (https://erasmusmcmgz.shinyapps.io/dissemination/).

## Results

### General patterns

**Fig 2:**
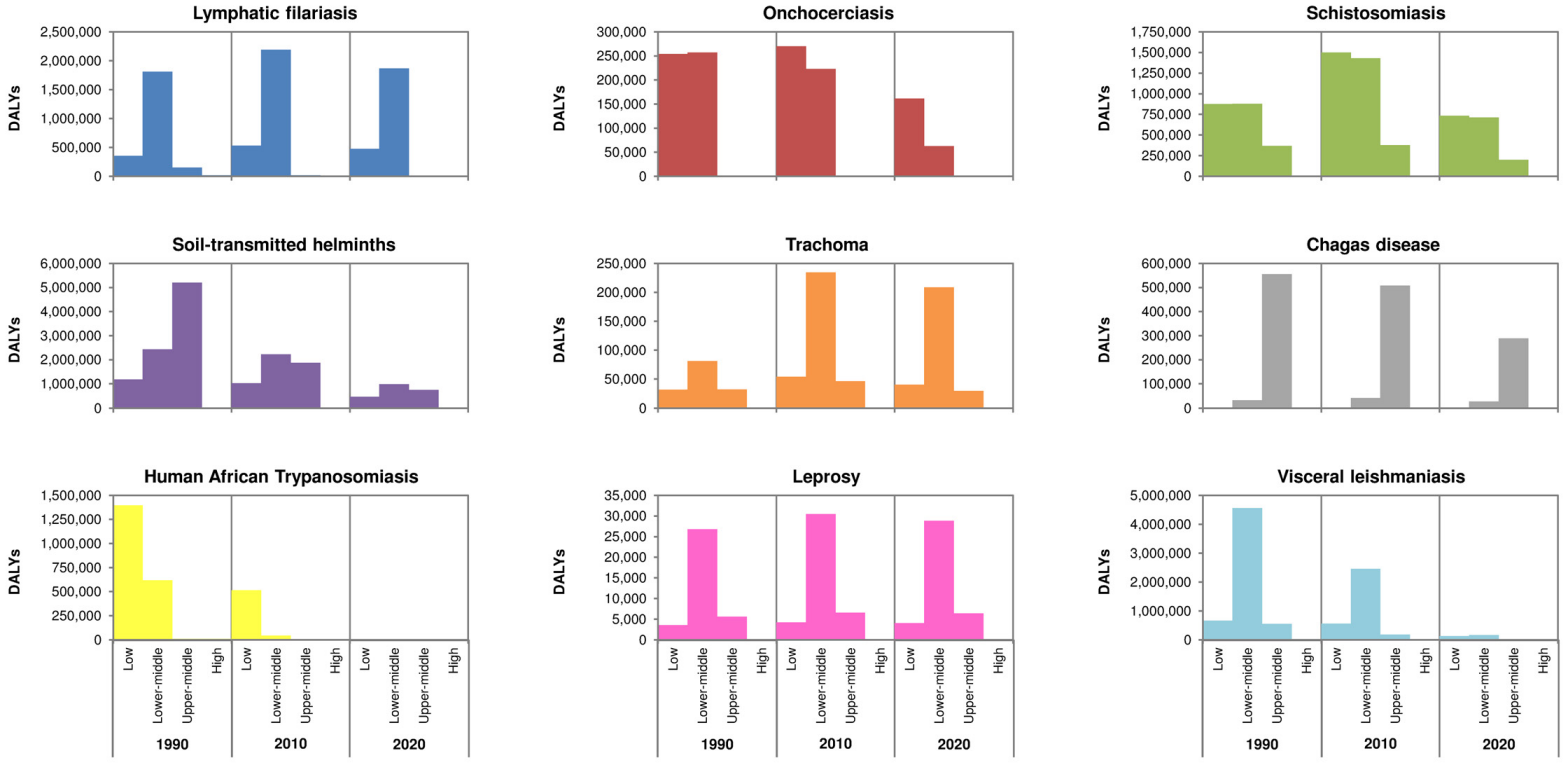
Burden of disease by country income group for nine NTDs, in 1990, 2010 and 2020.

Fig 2 summarizes the data, showing the absolute burden in DALYs per NTD by income group, for 1990, 2010, and 2020. The same information is numerically presented in Table 2. The burden of the nine NTDs is concentrated in low and lower-middle income countries. In 1990, low and lower-middle income countries held 43% of the world population, but accounted for 69% of the total burden caused by the 9 NTDs. For most of these NTDs, the share of the burden held by low and lower-middle income countries was even higher (around 80% for trachoma, schistosomiasis and leprosy; 90–100% for visceral leishmaniasis, lymphatic filariasis, onchocerciasis and human African trypanosomiasis). Only for the soil-transmitted helminthiases and Chagas disease, upper-middle income countries held a large share of the burden. By 2010, low and lower-middle income countries held 48% of the world population, but accounted for 81% of the global NTD burden.

**Table 2:** Burden of disease in 1990, 2010 and 2020, and relative change in 1990–2010 and 2010–2020.

		Burden of disease in DALYs, in thousands (%)			Relative change in disease burden^a^	
		1990	2010	2020	1990–2010	2010–2020
Lymphatic filariasis	Total	2,335 (100%)	2,736 (100%)	2,353 (100%)	17%	-14%
	Low	355 (15%)	528 (19%)	475 (20%)	49%	-10%
	Lower-middle	1,812 (78%)	2,189 (80%)	1,868 (79%)	21%	-15%
	Upper-middle	150 (6%)	17 (1%)	9 (0%)	-89%	-48%
	High	18 (1%)	1 (0%)	1 (0%)	-	-
Onchocerciasis	Total	512 (100%)	494 (100%)	224 (100%)	-4%	-55%
	Low	254 (50%)	270 (55%)	162 (72%)	6%	-40%
	Lower-middle	257 (50%)	223 (45%)	62 (28%)	-13%	-72%
	Upper-middle	0 (0%)	0 (0%)	0 (0%)	-	-
	High	1 (0%)	1 (0%)	0 (0%)	-	-
Schistosomiasis	Total	2,123 (100%)	3,308 (100%)	1,646 (100%)	56%	-50%
	Low	876 (41%)	1,500 (45%)	732 (44%)	71%	-51%
	Lower-middle	878 (41%)	1,429 (43%)	713 (43%)	63%	-50%
	Upper-middle	368 (17%)	376 (11%)	199 (12%)	2%	-47%
	High	2 (0%)	3 (0%)	2 (0%)	-	-
Soil-transmitted helminthiases	Total	8,816 (100%)	5,134 (100%)	2,200 (100%)	-42%	-57%
	Low	1,181 (13%)	1,026 (20%)	470 (21%)	-13%	-54%
	Lower-middle	2,431 (28%)	2,225 (43%)	980 (45%)	-8%	-56%
	Upper-middle	5,198 (59%)	1,875 (37%)	746 (34%)	-64%	-60%
	High	7 (0%)	7 (0%)	3 (0%)	-	-
Trachoma	Total	144 (100%)	334 (100%)	278 (100%)	133%	-17%
	Low	31 (22%)	54 (16%)	40 (14%)	73%	-26%
	Lower-middle	81 (56%)	234 (70%)	208 (75%)	190%	-11%
	Upper-middle	32 (22%)	46 (14%)	29 (11%)	45%	-36%
	High	0 (0%)	0 (0%)	0 (0%)	-	-
Chagas disease	Total	588 (100%)	550 (100%)	316 (100%)	-7%	-43%
	Low	0 (0%)	0 (0%)	0 (0%)	-	-
	Lower-middle	33 (6%)	42 (8%)	27 (9%)	27%	-35%
	Upper-middle	555 (94%)	508 (92%)	288 (91%)	-9%	-43%
	High	0 (0%)	0 (0%)	0 (0%)	-	-
Human African trypanosomiasis	Total	2,034 (100%)	560 (100%)	0 (100%)	-72%	-100%
	Low	1,396 (69%)	514 (92%)	0 (92%)	-63%	-100%
	Lower-middle	619 (30%)	44 (8%)	0 (7%)	-93%	-100%
	Upper-middle	10 (1%)	1 (0%)	0 (0%)	-	-
	High	9 (0%)	1 (0%)	0 (0%)	-	-
Leprosy	Total	36 (100%)	41 (100%)	39 (100%)	15%	-5%
	Low	4 (10%)	4 (10%)	4 (10%)	17%	-4%
	Lower-middle	27 (74%)	30 (74%)	29 (73%)	14%	-5%
	Upper-middle	6 (15%)	7 (16%)	6 (16%)	18%	-3%
	High	0 (1%)	0 (0%)	0 (0%)	-	-
Visceral leishmaniasis	Total	5,770 (100%)	3,198 (100%)	305 (100%)	-45%	-90%
	Low	659 (11%)	560 (18%)	125 (41%)	-15%	-78%
	Lower-middle	4,558 (79%)	2,455 (77%)	159 (52%)	-46%	-94%
	Upper-middle	548 (9%)	179 (6%)	20 (7%)	-67%	-89%
	High	4 (0%)	4 (0%)	0 (0%)	-	-
Total NTD	Total	22,358 (100%)	16,355 (100%)	7,362 (100%)	-27%	-55%
	Low	4,755 (21%)	4,458 (27%)	2,009 (27%)	-6%	-55%
	Lower-middle	10,695 (48%)	8,871 (54%)	4,047 (55%)	-17%	-54%
	Upper-middle	6,867 (31%)	3,009 (18%)	1,298 (18%)	-56%	-57%
	High	41 (0%)	17 (0%)	7 (0%)	-	-

^a^ Not calculated for an income group if the 1990 share of the disease burden was ≤1% in that income group

**Fig 3:**
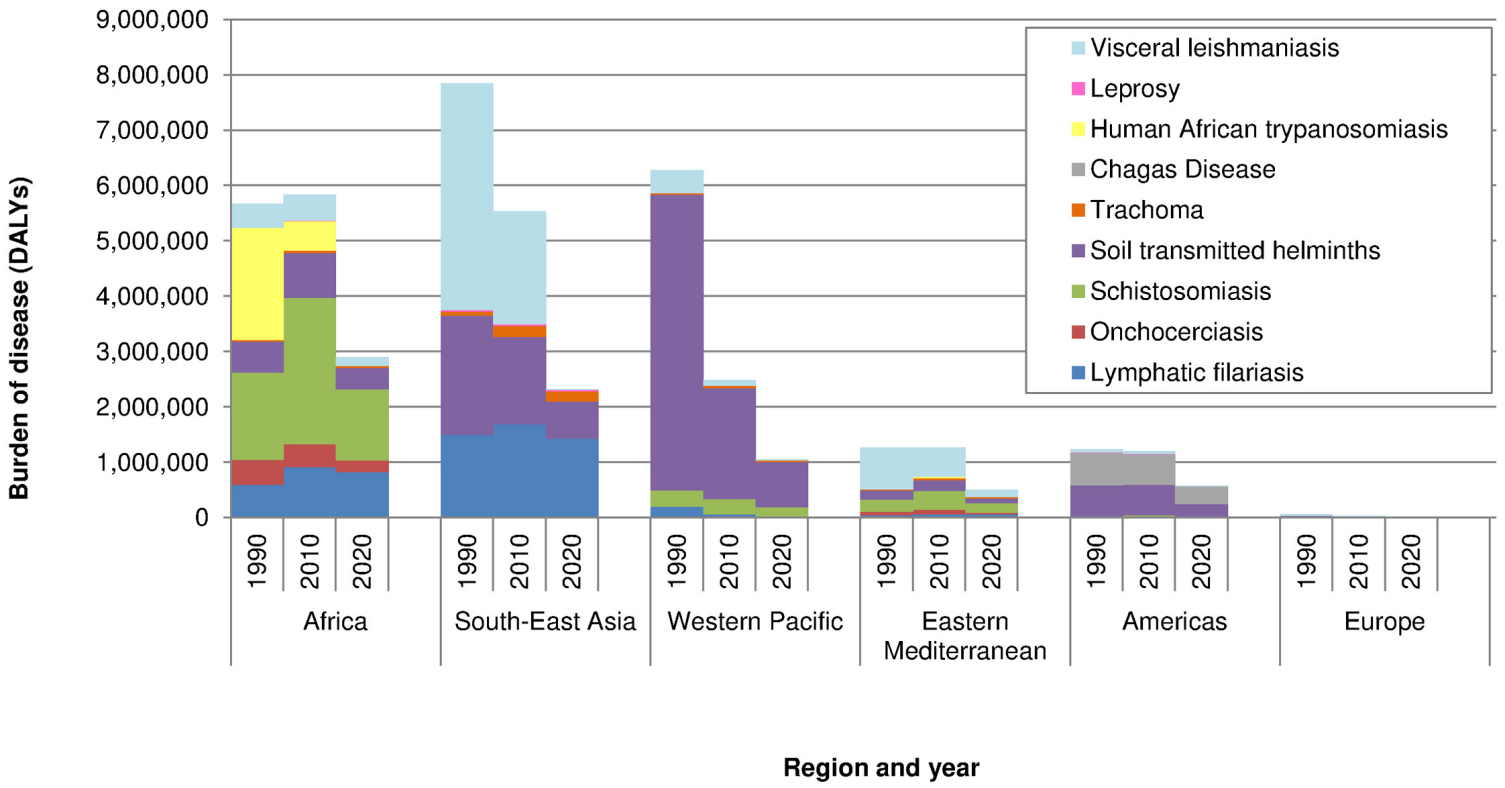
Burden of disease by NTD, region and calendar year.

The DALY distribution by income group largely intersects with the geographical distribution of the infections. Fig 3 shows the contribution of different diseases to the total burden from the nine NTDs by region and calendar year. S1 Fig presents the same data in a different way, highlighting how the burden is spread over regions, by disease and calendar year. Chagas disease is confined to Latin America; most countries in this region are classified as upper-middle income countries, explaining the peak in burden in this category. Diseases that predominantly occur in Africa (onchocerciasis, human African trypanosomiasis, and to a lesser extent schistosomiasis), were clustered in low and lower middle income countries, as 86% of the African population lives in such countries. Diseases that are particularly prominent in the South-East Asian region (lymphatic filariasis, visceral leishmaniasis, trachoma, leprosy), were clustered in the lower-middle income category. The soil-transmitted helminthiases are globally widespread, also in populous China, which was categorized as upper-middle income country, explaining the peak in burden in this income group in 1990.

**Fig 4:**
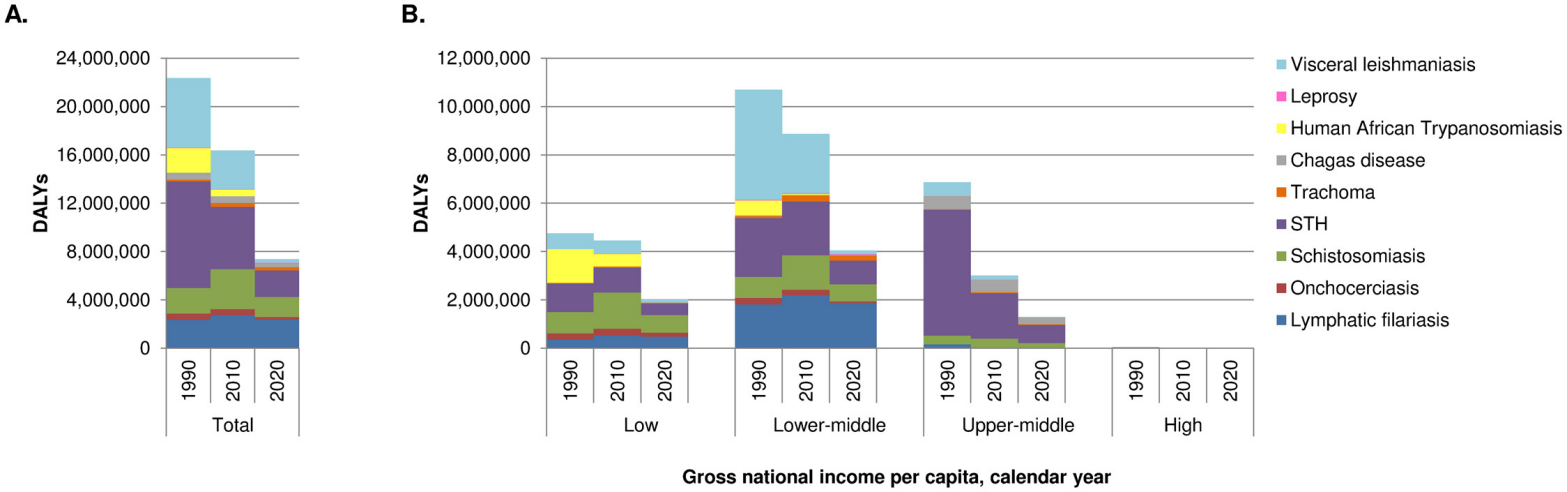
Time trend in the total burden of disease caused by the nine NTDs from 1990 to 2020, (A) globally and (B) by gross national income per capita.

Fig 4 shows the trend in disease burden overall and by country income group. Between 1990 and 2010, the global burden (in DALYs) of the nine NTDs declined by 27%. The decline varied strongly between income groups: the relative and absolute reduction was smallest in low income countries (6%; 297,000 DALYs) and greatest in upper-middle income countries (56%; 3,858,000 DALYs). Similar patterns were observed for the individual diseases. Between 1990 and 2010, the burden declined for five out of nine diseases, in particular for the soil-transmitted helminthiases, visceral leishmaniasis, and human African trypanosomiasis, with much smaller declines for onchocerciasis and Chagas disease (Table 2). For these diseases, the relative reduction was usually greater each step up the income hierarchy.

The 2020 burden was estimated under the assumption that the ambitious WHO roadmap targets would be met. For most diseases some burden remains in 2020, largely explained by chronic diseases that have developed from infections acquired in the past. Favorable exceptions are the soil-transmitted helminthiases (which are mainly associated with reversible conditions), visceral leishmaniasis and human African trypanosomiasis (for which the burden is largely caused by premature death soon after acquisition of the infection). While low income countries (followed by lower-middle income countries) profited least from improvements between 1990 and 2010, this is projected to be rectified in the 2010–2020 period. Achieving the WHO roadmap targets would imply a reduction in disease burden of around 55% in all country income groups (apart from a zero reduction in high income countries). In absolute terms, the reduction would be greatest in the low (2,449,000 DALYs) and lower-middle (4,824,000 DALYs) income groups. In total, 81% of the reduction that would be achieved in the 2010–2020 period, would be concentrated in low and lower-middle income countries, if the WHO roadmap targets were met.

For more detailed discussion of findings per disease, we refer to supplementary information in S1 Appendix. This appendix also provides additional information per disease on the 3–5 countries contributing most to the 2010 burden of disease, and the sequelae considered in the burden of disease estimates.

## Discussion

The burden of the nine NTDs under study is largely concentrated in low and lower-middle income countries. In 1990 and 2010, low and lower-middle income countries accounted for 42% and 48% of the world population respectively, but for about 69% and 81% of the global burden from these NTDs. For most of these NTDs, the burden of disease was even more strongly concentrated in low and lower-middle income countries; only for the soil-transmitted helminthiases and Chagas disease a considerable part of the burden occurred in upper-middle income countries. Between 1990 and 2010, the global burden of the nine NTDs in DALYs declined by 27%, which is largely attributable to declines in the soil-transmitted helminthiases, human African trypanosomiasis, and visceral leishmaniasis. The decline varied greatly between income groups: the reduction was as high as 56% in upper-middle income countries, but only 6% in low income countries, leading to a further concentration of the burden in low and lower-middle income countries. The slower adoption and implementation of recommended control strategies in the lowest income group may be related to multiple interrelated factors, including lack of human and financial resources, weak health systems, lack of donor support, societal unrest, and epidemiological factors. This underscores the importance of coordinated international programmes to control these NTDs worldwide and reduce the prevailing between-country health inequalities. Extra efforts from the international community will be needed to achieve the goals in the lowest income countries.

The 2020 burden was calculated under the assumption that the ambitious WHO roadmap targets would be met. If the targets would be met globally, the burden caused by the nine selected NTDs is expected to decline by about 55% in all income groups (apart from high income countries) between 2010 and 2020. In absolute terms, the decline would be largest in the lower-middle income group, because this group holds the largest share of the 2010 burden. 81% of the reduction that would be achieved in the 2010–2020 period, would occur in low and lower-middle income countries. Some burden would still remain by 2020, because the burden largely arises from chronic diseases that have developed from infections acquired in the past. The targets are highly ambitious and may not be met everywhere with current strategies, e.g. due to a late start of slow scale-up of interventions [19, 20], epidemiological circumstances that require more intensive and/or longer duration of interventions [21–23], or failure to reach everyone with interventions [21]. Yet, by intensifying efforts or adopting modified strategies, it may still be possible to reach the targets in many settings, thereby achieving the important impacts described in the current paper and other papers in this series.

We performed an ecological analysis. Care is required when making inferences regarding the association between poverty and NTDs at the individual level. For example, our analysis shows that a non-negligible part of the NTD burden in 1990 and 2010 occurred in upper-middle income countries (classified based on 2010 GNI per capita). It should be realized, though, that there can be large within-country differences in income and health within the highly populous upper-middle income countries like China and Brazil. Within these countries, the actual burden of NTDs is arguably still concentrated in the poorest subgroups, so that the clustering in the poor might even be stronger than suggested here. To get a more complete picture of the clustering of NTDs among the poor, we have also reviewed published literature on within-country differences in prevalence of NTDs. Although the amount and strength of evidence varied somewhat between diseases, overall there is considerable evidence that socioeconomically disadvantaged groups have higher infection rates [24].

We based our analysis on the disease burden estimates as calculated in the GBD 2010 study [1]. The absolute burden estimates are notably uncertain for NTDs, due to lack of data on their geographic spread and control, uncertainties about the association between acute or chronic infection and specific morbidities, and uncertainties about assigned disability weights [2]. Most GBD 1990 and 2010 estimates for NTDs show very wide confidence intervals, often ± 50% of the mean, but sometimes with an upper confidence limit up to 5 times the mean. Although considerable uncertainty exist with respect to absolute disease burden estimates for specific countries, this will probably not have strong impact on our estimates of the share of the burden in country income groups, which primarily depend on the geographic spread of the various NTDs and the distribution of the world population over the different income strata.

This paper concentrates on 9 diseases named in the London Declaration on NTDs (excluding Guinea worm), but there are many more. In recent reports, WHO has categorized 17 diseases as NTDs, including the endemic treponematoses (yaws), human dog-mediated rabies, dengue, buruli ulcer, cutaneous leishmaniasis, taeniasis/cysticercosis and echinococcosis/hydatidosis, foodborne trematode infections. The 9 diseases considered here together constituted about 63% of the global burden caused by the 17 NTDs listed by WHO, according to the GBD 2010 study, but this proportion is almost halved if the definition of NTDs is broadened to include also other NTDs [2]. The burden caused by these other NTDs can be similarly high as from the other diseases. As yet these diseases do not have the same opportunities for control and elimination due to unavailable or compromised tools, and WHO targets include a need to validate strategies and perform pilot projects. Eventually, though, these NTDs also need to be addressed and mitigated.

We did not assess the poverty-impact of these measures. This impact is probably is clearest if catastrophic health expenditures are prevented, e.g. for human African trypanosomiasis [14]. We should acknowledge the possibility that the impact of these interventions on the total disease burden, also due to other causes, might to some extent be attenuated because competing causes of morbidity and death might replace the NTDs. This would also attenuate the immediate impact of these interventions on poverty. Nevertheless, affected populations will be left in a better position to escape from the poverty trap.

NTDs as a group are known to constitute a major health burden, which is largely concentrated in low and lower-middle income countries. Between 1990 and 2010, the disease burden from NTDs has declined considerably in upper-middle income countries, but hardly declined in the low income countries. Achieving internationally agreed targets of NTD control and elimination will bring about major gains in health and reductions in human suffering, with 81% of the global reduction occurring in low and lower-middle income countries. This would reduce the prevailing between-country health inequalities.

## Supporting Information

S1 Table Interpretations of WHO Roadmap targets as used in our calculations. (DOCX)

S1 Datafile data underlying all calculations (XLSX)

S1 Fig Burden of disease by NTD, WHO Region and year, A) in absolute terms, B) as proportion of the global burden per NTD.
(DOCX)

S1 Appendix Distribution of NTD disease burden over country income groups, highlighting the countries contributing most to the 2010 disease burden, for each NTD separately. (DOCX)
