# Supplementary material for: Between-Country Inequalities in the Neglected Tropical Disease Burden in 1990 and 2010, with Projections for 2020
Source: PLoS Negl Trop Dis. 2016 May 12;10(5):e0004560. doi: 10.1371/journal.pntd.0004560 (PMC4865216; doi:10.1371/journal.pntd.0004560)
Supplement: S1 Table — (DOCX) [file pntd.0004560.s001.docx]

**S1 Table. Interpretations of WHO Roadmap targets [1] as used in our calculations.** All country-specific assumptions for each NTD are provided here: <https://erasmusmcmgz.shinyapps.io/dissemination/>

| Lymphatic filariasis | **Target:** By 2020, 70% of countries will have been verified as free of transmission and 30% will have entered post-intervention surveillance.  **Interpretation:** The PCT database [2] provides start and end years of the intervention program per country. The incidence of both chronic manifestations (lymphedema and hydrocele) is assumed to linearly decrease to zero, one year before the anticipated last treatment round in each country. |
| --- | --- |
| Onchocerciasis | **Target:** To eliminate onchocerciasis where feasible (without a specified target year).  **Interpretation:** Interventions will continue until the end year of interventions as estimated by APOC [3]; prevalence of skin disease and incidence of vision loss reach zero two years before the end year of interventions. |
| Schistosomiasis | **Target:** Elimination of transmission in certain regions and countries by 2015 or 2020. Global elimination in 2025 as a public health problem. In 2020, 75% national coverage is reached in all the countries requiring preventive chemotherapy for schistosomiasis.  **Interpretation:** Global elimination in 2025, therefore in all countries prevalence of reversible and incidence of irreversible sequelae will go down to zero in 2025. The general start year of interventions is 2001, the same year as the WHA resolution on STH and schistosomiasis [4]. |
| STH  (ascariasis, hookworm disease and trichuriasis) | **Target:** 100% of countries requiring preventive chemotherapy for STH have achieved 75% national mass drug administration coverage of school-aged children (SAC) and pre-SAC by 2020.  **Interpretation:** The pre-SAC and SAC (ages 5-14) will have 0% prevalence of morbidity by 2025. There will be 10% remaining prevalence of morbidity in the non-treated groups (0-4 and 15+) by the year 2025, relative to the STH level in 2010. Mortality due to ascariasis will be 0% in 2025, for all age groups. The general starting year of interventions is 2001, the same year as the WHA resolution on STH and schistosomiasis [4]. |
| Trachoma | **Target:** Global elimination as a public health problem in 2020. All countries will have achieved the ultimate intervention goal and be free from blinding trachoma as a public health problem.  **Interpretation:** Incidence of vision loss caused by trachoma will go down to zero in country specific years. Three WHO documents [1,5,6] provide most start years of intervention and target years of elimination. |
| Chagas’ disease | **Target:** To eliminate transmission through blood transfusion in the America’s, Europe and Western Pacific by 2015. To eliminate peri-domiciliary infestation in Latin America by 2020, but surveillance and control of oral transmission and congenital infection need to be sustained.  **Interpretation:** For acute Chagas’ disease the prevalence will linearly decrease to 10% of the GBD 2010 value in 2020, and remain 10% onwards. This 10% reflects remaining burden due to infections from the sylvatic cycle. For chronic heart disease, chronic digestive disease and heart failure, incidence in 2020 will be 10% of that in 2010, and remain 10% onwards. The GBD data about Chagas’ disease only concern countries in Latin America, so no specific assumptions are needed for the rest of the world.  **Note:** There are great concerns about the reliability of the GBD figures, as well as the feasibility of the London Declaration and associated WHO targets [7]. |
| HAT | **Target:** Achieve elimination of >90% of foci by 2020. The global number of new cases reported annually for 2020 is <2000.  **Interpretation:** There are exactly 2000 cases in 2020, and this number will subsequently decline to 0 in 2030. From 2020 onwards, all remaining cases will be detected and treated, meaning that HAT mortality is zero in 2020 and beyond. The overall number of prevalent cases in 2020 is 2.5% of the level in 2010.  **Explanation:** In 2010, 9103 people died because of HAT according to YLL data of GBD 2010. By multiplying the number of people that died in 2010 by 3 (the burden before dying is assumed to last on average for three years in the GBD calculations) we arrive at 27,307 prevalent cases in 2010 that will eventually die because of HAT. The total point prevalence of HAT in 2010 is provided by the GBD: 36,863. This means that about 75% (27,307 out of 36,863) of prevalent cases will eventually die, whereas the remaining 25% (9,554 out of 36,863) will survive. The 9,554 surviving prevalence cases multiplied by 2 results in 19,108 new detected and successfully treated cases in 2010 (given the GBD assumption that disease lasts 6 months before treatment). Thus, on a global level there will be a decrease from 19,108 new surviving cases in 2010 down to 2000 new surviving cases in 2020, so roughly a decrease to 10% of the level in 2010. This decrease applies to the number of surviving prevalent cases, which is 25% of the total. This means that the overall number of prevalent cases will go down to 10% times 25% = 2.5% of the level in 2010, as the 75% of cases that eventually die will become 0.  **Note:** The number of new detected and treated cases (19,108) and the number of new cases that will die (9,554) adds up to 28,211 new cases in 2010, which is substantially higher than what is known in WHO records [8]: 7139 new reported cases in 2010. |
| Leprosy | **Target:** Global interruption of transmission by 2020. Reduction of grade 2 disabilities in newly detected cases to below 1/million population at global level by 2020.  **Interpretation:** The incidence of disfigurement due to leprosy has decreased in 2020 to 37% of the level in 2010, and will further reduce to 0% in 2030, in order to account for the target of global interruption of transmission by 2020.  **Explanation:** According to the 2010 GBD data, the incidence of all newly detected cases was 318,876, of which 6% (19,132) had grade 2 disability [9]. This is 2.7/1 million globally. According to the WHO target, this should be reduced to 1/1 million in 2020, representing a reduction to approximately 37% of the level in 2010, or 7,086 incident cases in 2020. |
| Visceral leishmaniasis | **Target:** On the Indian subcontinent (ISC), 1/10,000 new cases at (sub)district level per year by 2020; globally, 100% detection and treatment of VL.  **Interpretation:** On ISC there will be a prevalence reduction to 5% of the 2010 situation, which will remain at 5% until 2030. Elsewhere, the prevalence of 2010 will remain unaltered. Morbidity in 2020 will have become 25% (Africa), 0.3% (ISC) and 10% (elsewhere) of the level in 2010, and remain constant thereafter.  **Explanation about prevalence on ISC**: WHO reports approximately 20/10,000 new VL cases per year on ISC in 2010. Therefore, the target of 1/10,000 will be a reduction to 5% of the 2010 situation. This 5% will also apply to the prevalent cases.  **Explanation about trends in death:** In 2010, 51,485 people died because of VL according to YLL data of GBD 2010. Also, worldwide there were 67,721 prevalent cases, which correspond to 270,884 new cases, given the GBD-assumed 3 month average duration of VL. Thus, in 2010 on average 19% of the people with VL died globally. According to the WHO targets, death due to VL will decrease substantially, but it will not go down to zero, as current treatment is not 100% effective [10]. We assume that in Africa 5% of the people (even though detected and treated) with VL will die in 2020, 1% of the people with VL on the Indian subcontinent, and 2% elsewhere. This means that in Africa the relative number of deaths (and also YLL) will decrease to 5/19 = about 25% of the level in 2010. On the Indian subcontinent this will be 0.05 times 1/19 = about 0.3% of the level in 2010. Elsewhere, this will be 2/19 = about 10% of the level in 2010. The regional differences in mortality rates were based on discussions with the disease experts and particularly reflect differences in treatment efficacy and HIV-coinfection. |

**References**

1. World Health Organization (2012) Accelerating work to overcome the global impact of neglected tropical diseases: a roadmap for implementation (<http://www.who.int/neglected_diseases/NTD_RoadMap_2012_Fullversion.pdf>; accessed 16 March 2015).
2. World Health Organization, PCT databank (<http://www.who.int/neglected_diseases/preventive_chemotherapy/lf/en/>; accessed 7 April 2015).
3. African Programme for Onchocerciasis Control (WHO/APOC) (2013). Revised plan of action and budget 2014–2015: Elimination of onchocerciasis in Africa. JAF document 19.9 (November 2013)

(<http://www.who.int/apoc/about/structure/jaf/Final_Communique_JAF19_Final_English_140114.pdf?ua=1>; accessed 7 April 2015).

1. World Health Assembly (2001) WHA 54.19, Schistosomiasis and soil-transmitted helminth infections (<http://www.who.int/neglected_diseases/mediacentre/WHA_54.19_Eng.pdf>; accessed 7 April 2015).
2. World Health Organization (2013) Global Alliance for the Elimination of Blinding Trachoma by 2020: Progress report on elimination of trachoma, 2012. Wkly Epidemiol Rec 88, 242–251 (<http://www.who.int/wer/2013/wer8824.pdf?ua=1>; accessed 7 April 2015).
3. World Health Organization (2013) Report of the 17^th^ meeting of the WHO alliance for the global elimination of blinding trachoma. Geneva, 22-24 April 2013 (<http://www.who.int/blindness/publications/GET17Report_final.pdf?ua=1>; accessed 10 April 2015).
4. Tarleton RL, Gürtler RE, Urbina JA, Ramsey J, Viotti R (2014) Chagas disease and the London Declaration on neglected tropical diseases. PLoS Negl Trop Dis 8: e3219. doi:10.1371/journal.pntd.0003219
5. World Health Organization, Human African trypanosomiasis: The current situation (<http://www.who.int/trypanosomiasis_african/country/country_situation/en/>; accessed 7 April 2015).
6. World Health Organization (2011) Leprosy update, 2011. Wkly Epidemiol Rec 86, 389–399.
7. Moore EM, Lockwood DN (2010) Treatment of visceral leishmaniasis. J Global Infect Dis 2: 151–158.
