# Supplementary material for: Between-Country Inequalities in the Neglected Tropical Disease Burden in 1990 and 2010, with Projections for 2020
Source: PLoS Negl Trop Dis. 2016 May 12;10(5):e0004560. doi: 10.1371/journal.pntd.0004560 (PMC4865216; doi:10.1371/journal.pntd.0004560)
Supplement: S1 Appendix — (DOCX) [file pntd.0004560.s004.docx]

**Lymphatic filariasis**

**Distribution of burden over country income groups, highlighting the countries contributing most to the 2010 disease burden:**

**Findings:**

- Distribution of disease in 1990: Over 93% of the burden was concentrated in low and lower-middle income countries. Three populous countries accounted for about 70% of the burden, i.e. India (57%), Nigeria (8%) and Indonesia (5%).
- Change in burden between 1990 and 2010: The burden in low income countries showed a strong increase (almost 50%), mainly due to population growth. Lower-middle income countries experienced a less pronounced increase (21%), while the burden in upper-middle and high income countries almost completely disappeared (reduction of about 90%). As a result, low and lower middle income countries accounted for 99% of the burden in 2010. The three countries contributing most to the 2010 burden were India (55%), Nigeria (10%) and Indonesia (4%).
- Impact of meeting the WHO targets for 2020: Even with a successful interruption of transmission, the reduction in disease burden between 2010 and 2020 would be relatively modest (-14%). This is explained by the chronic nature of disability associated with LF. We do not take into account the impact of morbidity control measures, which are not systematically rolled out with preventive chemotherapy.

**Notes:**

- Sequelae considered in GBD study: lymphedema and hydrocele (both are assumed to be irreversible)

**Onchocerciasis**

**Distribution of burden over country income groups, highlighting the countries contributing most to the 2010 disease burden:**

**Findings:**

- Distribution of disease in 1990: The burden of onchocerciasis was concentrated in the low and lower-middle income countries within the African region. Small foci existed in Latin America and Yemen. Low and lower-middle income countries account for 50% of the burden each. The geographic spread was primarily determined by the presence of suitable breeding sites for the black fly vector. The three countries contributing most to the total burden were Democratic Republic of Congo (28%), Nigeria (24%) and Sudan (11%), all belonging to the most populous countries on the African continent.
- Change in burden between 1990 and 2010: Onchocerciasis has a long history of control and the total burden has declined. The impact largely came to the benefit of lower-middle income countries. By 2010, Democratic Republic of Congo, Nigeria and Sudan were still the main contributors to the burden, but DRC accounted for almost 40% of the burden, Nigeria still for 20% and Sudan also for 16%. The relative contribution of Democratic Republic of Congo and Sudan have increased, because these countries were lagging behind in the implementation of control activities.
- Impact of meeting the WHO targets: Strong impact expected by 2020: between 2010 and 2020 the total burden would be reduced by 55% if the WHO targets are met. However, due to differences in the history of control (start year of interventions, rate of scaling up) the impact varies between countries. By 2020 many countries will already have eliminated transmission and the burden of chronic disease will show a considerable decline in these countries. In other countries (such as Central African Republic, Cameroon, Democratic Republic of Congo) interventions will need to continue beyond 2020 to achieve the same. Least progress would be achieved for Democratic Republic of Congo (due to delayed start of interventions in combination with highly unfavourable transmission dynamics), and by 2020 this country accounts for over 60% of the global burden.

**Notes:**

- Sequelae considered in GBD study: visual impairment, blindness (both irreversible) and skin manifestations with and without itch (mostly reversible)

**Schistosomiasis**

**Distribution of burden over country income groups, highlighting the countries contributing most to the 2010 disease burden:**

**Findings:**

- Distribution of disease in 1990: Sub Sahara Africa accounted for almost 80% of the global burden of schistosomiasis, but the disease also occured in South America, the Caribbean, the Middle East, and East Asia. Upper-middle income countries accounted for almost 20% of the global burden, while the remaining 80% was evenly spread among low and lower-middle income countries. The top three burden countries in 1990 were Nigeria (20%), China (13%) and Ethiopia (7%).
- Change in burden between 1990 and 2010: The GBD data suggest a large increase in burden between 1990 and 2010. This can only partly be explained by population growth, and may also reflect an underestimation of the 1990 burden. Nigeria, China and Ethiopia were still contributing most to the global burden (21%, 8% and 7% respectively). The distribution over income groups did not change much in this period.
- Impact of meeting the WHO targets: Elimination of schistosomiasis as a public health problem is to be achieved by 2025 globally. Between 2010 and 2020, the global burden would be halved. Nigeria, China and Ethiopia would still contribute most to the global burden, accounting for similar shares as in 2010.

**Notes:**

- Sequelae considered in GBD study: schistosomiasis infestation, mild diarrhea, hematemesis, bladder pathology, dysuria, hepatomegaly, hydronephrosis, ascites, anemia

**Soil-transmitted helminths**

**Distribution of burden over country income groups, highlighting the countries contributing most to the 2010 disease burden:**

**Findings:**

- Distribution of disease in 1990: As a group, the three STHs (ascariasis, hookworm, and trichuriasis) were the most important cause of disability among the NTDs. STH is globally widespread, occurring in low, lower-middle and upper-middle income countries. In 1990, upper-middle income countries accounted for almost 60% of the global burden; low and lower-middle income countries accounted for 13% and 28% of the global burden respectively. In 1990, China accounted for 50% of the global burden. Other major contributors were India (10%), Bangladesh (6%), Philippines (4%) and Vietnam (4%).
- Change in burden between 1990 and 2010: The global burden declined by 42%, but this was unevenly distributed across income groups. While the burden in low and lower middle income countries declined by around 10%, the burden in upper middle income countries declined by about two-thirds thanks to the strong reduction in China. In 2010, lower middle income countries accounted for the largest share of the burden (43% versus 37% in upper middle and 20% in low income countries). China, India, Bangladesh, Philippines and Vietnam were still contributing most to the global burden (20%, 16%, 7%, 7% and 7%, respectively).
- Impact of meeting the WHO targets: Meeting the STH 2020 targets would result in a 57% reduction in the global burden compared to the 1990 level, and the relative reduction does not differ much between income groups.

**Notes:**

- Sequelae considered in GBD study: All three STH (ascariasis, hookworm, trichuriasis) are associated with infestation, mild abdominopelvic problems and – in children <5 – severe wasting. Hookworm causes anemia in addition. Ascariasis is associated with excess mortality.

**Trachoma**

**Distribution of burden over country income groups, highlighting the countries contributing most to the 2010 disease burden:**

**Findings:**

- Distribution of disease in 1990: Trachoma is endemic in the poorest regions of Africa, Asia, and the Middle East and in some parts of Latin America and Australia. It occurs in low, lower-middle and upper-middle income countries. In 1990, the lower-middle income group accounted for 56% of the burden, with India as main contributor (46% of the global burden); low and upper-middle income countries each accounted for 22% of the burden. Other high-burden countries were China (18%), Ethiopia (7%) and Pakistan (6%).
- Change in burden between 1990 and 2010: The GBD data suggest a large increase in burden from 1990 to 2010. This can only partly be explained by population growth, and may reflect an underestimation of the 1990 burden. India, China, Pakistan and Ethiopia were still contributing most to the global burden (58%, 12%, 9% and 6% respectively).
- Impact of meeting the WHO targets: Although the transmission should be interrupted by 2020, the associated reduction in the burden of disease between 2010 and 2020 would be relatively modest (-17%). This is explained by the chronic nature of disability: although no new cases of low vision and blindness are expected, existing cases and the associated burden of disease will only disappear slowly. The distribution over income groups remains more or less constant.

**Notes:**

- Sequelae considered in GBD study: low vision and blindness (both irreversible)

**Chagas disease**

**Distribution of burden over country income groups, highlighting the countries contributing most to the 2010 disease burden:**

**Findings:**

- Distribution of disease in 1990: Unlike most other NTDs, Chagas disease is concentrated mostly in upper-middle income countries. This is caused by regional clustering: the disease is restricted to the Latin American and Caribbean region, where most countries are in the upper-middle income group. In 1990, the Chagas disease burden distribution over income groups within the American region largely reflected the population distribution. The three countries contributing most the total burden in 1990 were Brazil (56%), Mexico (14%) and Argentina (7%).
- Change in burden between 1990 and 2010: The GBD data indicate that there was a small reduction in the overall disease burden, which was unevenly distributed between income groups: the burden was reduced (‑9%) in upper-middle income countries, while the burden in lower-middle income countries increased (+27%). Brazil, Mexico and Argentina remained the top three contributing countries to the 2010 burden.
- Impact of meeting the WHO targets for 2020: the burden of disease would be reduced by 43%. A considerable burden would remain because the clinical manifestations of Chagas are mostly chronic and existing cases are not cured by the interventions. Also, the infection would not be eliminated completely by 2020.

**Notes:**

- Sequelae considered in GBD study: acute chagas disease (reversible), chronic heart disease (irreversible), chronic digestive disease (irreversible), heart failure due to Chagas’ disease (irreversible)
- Data only capture burden in endemic countries of Latin America and the Caribbean and not the burden in migrants who now live in other regions
- There are great concerns about the reliability of these data, which are based on limited data

**Human African trypanosomiasis**

**Distribution of burden over country income groups, highlighting the countries contributing most to the 2010 disease burden:**

**Findings:**

- Distribution of disease in 1990: Of all NTDs included in the study, HAT shows the highest concentration in low income countries. The disease occurs only in Sub Sahara Africa, and within this region is it concentrated in the low and lower-middle income countries. The geographic distribution is directly linked to the range of its vector, the tse tse fly. By 1990, the lowest income countries held 53% of the regional population, but almost 70% of the burden. The three countries contributing most to the total burden were Democratic Republic of Congo (30%), Uganda (24%) and Ghana (12%).
- Distribution of impact of control between 1990 and 2010: The overall burden of disease was reduced by over 70%, but the impact was unevenly distributed over income groups: the burden in lower middle income countries was reduced by 93% and the relative reduction was similarly high in upper middle and high income countries. However, the reduction in low income countries was only 63%. By 2010 the disease almost exclusively appears in low income countries. By 2010, Democratic Republic of Congo, Central African Republic and Chad were most affected, holding respectively 52%, 16% and 14% of the total HAT burden.
- Impact of meeting the WHO targets for 2020: If the WHO targets for 2020 were met, the burden of disease would almost completely disappear because the infection would be eliminated in 90% of the foci, and remaining cases would be detected and successfully treated.

**Notes:**

- Sequelae considered in GBD study: human african trypanosomiasis, leading to death if not successfully treated
- The number of new detected and treated cases (19,108) and the number of new cases that will die (9,554) adds up to 28,211 new cases in 2010. This number is clearly a substantial overestimation of what is known in WHO records: 7139 new reported cases in 2010.

**Leprosy**

**Distribution of burden over country income groups, highlighting the countries contributing most to the 2010 disease burden:**

**Findings:**

- Distribution of disease in 1990: In 1990, leprosy occured in low, lower-middle and upper-middle income countries, the lower-middle category holding ¾ of the total burden. India accounted for 62% of the 1990 burden, Brazil for 12% and Indonesia for another 5%.
- Change in burden between 1990 and 2010: The overall burden of disease increased by about 15% between 1990 and 2010, and the relative increase did not differ much between income strata.
- Impact of meeting the WHO targets for 2020: Although the transmission would be interrupted by 2020, this would not be reflected immediately in the detection rate of new cases with grade 2 disability due to the long incubation period. Due to the chronic nature of disability and the delayed impact on the new cases detection rate the reduction in the burden of disease between 2010 and 2020 would be relatively modest.

**Notes:**

- Sequelae considered in GBD study: grade 2 disability due to leprosy
- The GBD 2010 estimates for leprosy appeared to be mistakenly based on overall leprosy new case detection (incident cases) instead of prevalence of (irreversible) cases with leprosy grade 2 disability, on which the disability weights are based. We therefore performed a recalculation to arrive at grade 2 disability prevalences as follows as explained in the main paper of this series by De Vlas et al: De Vlas SJ, Stolk WA, Le Rutte EA, Hontelez JAC, Bakker R, Blok DJ, et al. Concerted efforts to control or eliminate neglected tropical diseases: how much health will be gained? PLoS negl Trop Dis (submitted, paper 1 of this collection))..

**Visceral leishmaniasis**

**Distribution of burden over country income groups, highlighting the countries contributing most to the 2010 disease burden:**

**Findings:**

- Distribution of disease in 1990: visceral leishmaniasis was highly endemic in the Indian subcontinent and in East Africa, but also occured in other regions. The lower-middle income group accounted for 79% of the burden; low, upper-middle and high income countries respectively accounted for 11%, 9% and 0.1% of the global burden. India (lower middle income) accounted for 66% of the global burden. Other important contributing countries were China (7%), Sudan (5%), Bangladesh (4%), Egypt (4%) and Ethiopia (2%).
- Change in burden between 1990 and 2010: Between 1990 and 2010, the global burden declined by 45%. The relative reduction however was smallest in low income groups (15% reduction), was equal to the global level in the lower-middle income group (46% reduction), and highest in the upper-middle income group (67% reduction). India (59%), Sudan (7%), Bangladesh (4%), Egypt (4%), Ethiopia (3%), China (3%) were still the main contributors, although China was no longer the 2nd contributor.
- Impact of meeting the WHO targets: A dramatic decline in the burden would be expected if the WHO targets were met: the burden in India would be as good as eliminated, and that in other countries considerably reduced because all new cases would be treated. Since treatment is not 100% effective, this would not eliminate the total burden. We assume that in Africa still 5% of the people with visceral leishmaniasis (even though detected and treated) would die in 2020; this percentage would be less than 1% of the people with visceral leishmaniasis on the Indian subcontinent, and 2% elsewhere.

**Notes:**

- Sequelae considered in GBD study: visceral leishmaniasis, leading to death if left untreated and in some percentage of treated patients.
