# Supplementary material for: Between-Country Inequalities in the Neglected Tropical Disease Burden in 1990 and 2010, with Projections for 2020
Source: PLoS Negl Trop Dis. 2016 May 12;10(5):e0004560. doi: 10.1371/journal.pntd.0004560 (PMC4865216; doi:10.1371/journal.pntd.0004560)

Supporting Information – S1 Figure: Burden of disease by NTD, WHO Region and year, A) in absolute terms, B) as proportion of the global burden per NTD.

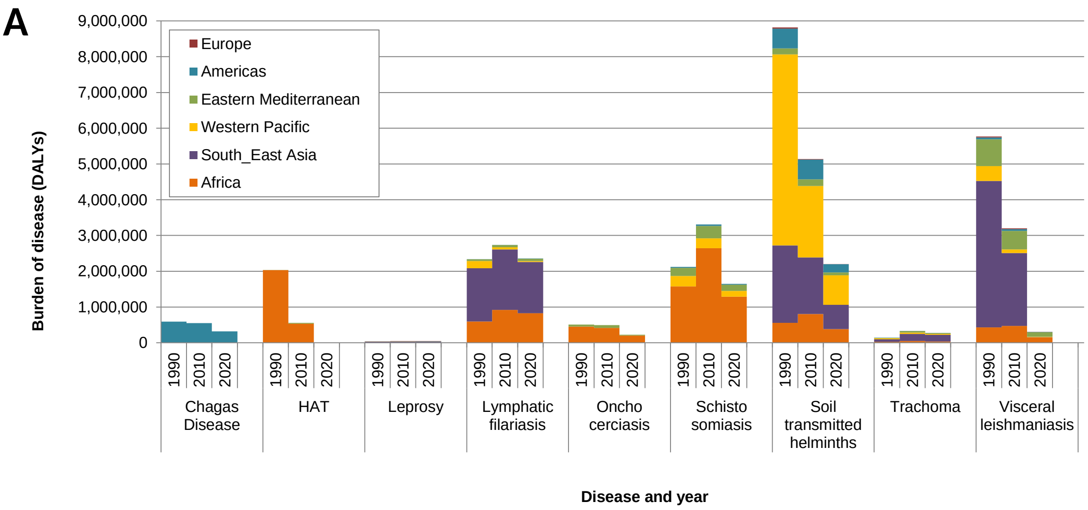

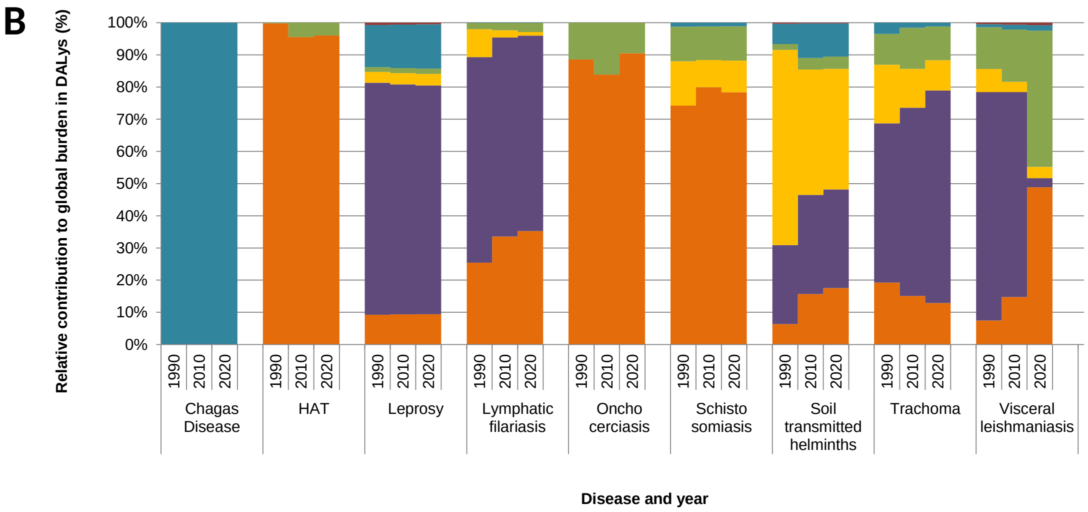

Supplement: Supplementary file 4 [file pntd.0004560.s003.docx]
